# Risk factors of rotavirus diarrhea in hospitalized children in Sanglah Hospital, Denpasar: a prospective cohort study

**DOI:** 10.1186/1471-230X-14-54

**Published:** 2014-03-26

**Authors:** Hendra Salim, I Putu Gede Karyana, I Gusti Ngurah Sanjaya-Putra, Soetjiningsih Budiarsa, Yati Soenarto

**Affiliations:** 1 Division of Gastroenterology and Hepatology, Department of Child Health, Medical School, Udayana University/Sanglah Hospital, Denpasar, Indonesia; 2 Division of Child Growth and Development – Social Pediatrics, Department of Child Health, Medical School, Udayana University/Sanglah Hospital, Denpasar, Indonesia; 3 Division of Gastroenterology and Hepatology, Department of Child Health, Medical School, Gadjah Mada University/Sardjito Hospital, Yogyakarta, Indonesia

**Keywords:** Acute diarrhea, Rotavirus, Children

## Abstract

**Background:**

Diarrhea is a major public health concern throughout the world because the prevalence of morbidity of diarrhea has not changed significantly in the past decade. It remains the third leading cause of death among children less than 5 years of age. Recent surveillance studies have shown that rotavirus is a significant cause of pediatric hospitalization and death due to diarrhea. Indonesia has limited data on risk factors, disease burden, and deaths in children due to rotavirus diarrhea. The objective of this study was to examine the above mentioned factors related to rotavirus diarrhea in hospitalized children in Sanglah Hospital, Denpasar.

**Methods:**

A prospective cohort study was conducted at Sanglah Hospital Denpasar from April 2009 to December 2011. The present study was part of a nationwide study on Extension for Hospital-based Surveillance and Strain Characterization of Rotavirus Diarrhea Indonesia involving four hospitals throughout Indonesia as a part of the Asian Rotavirus Surveillance Network. We studied children aged <5 years who were hospitalized with acute diarrhea, and analyzed their stool samples using an immunoassay that detects the rotavirus antigen.

**Results:**

A total of 656 patients met the inclusion criteria for this study. Of 5805 patients under the age of 5 who were hospitalized between April 2009 and December 2011, the prevalence of diarrhea among hospitalized pediatric patients was 11.3% and the prevalence of rotavirus diarrhea was 49.8%. The male to female ratio of those affected by rotavirus was 1.6:1. The occurrence of vomiting was significantly higher in rotavirus diarrhea than in non-rotavirus diarrhea (RR, 1.4; 95% CI, 1.08 to 1.70; p = 0.004).

**Conclusions:**

Diarrhea remains an important cause of hospitalization in children, and rotavirus was the most important etiology. We found that boys had a greatest risk of rotavirus infection than girls. Good nutritional status and breastfeeding provided the same protection against rotavirus and non-rotavirus diarrhea.

## Background

Rotavirus is recognized as a major cause of non-bacterial gastroenteritis (infection of the stomach and intestinal tract leading to diarrhea and vomiting) especially in infants and young children worldwide [1,2]. Rotavirus gastroenteritis is endemic worldwide. The infection is associated with high rate of morbidity throughout the world and a high rate of mortality in developing countries. It account for more than 800,000 child deaths per year due to poor nutrition and health care in developing countries [3].

Diarrhea has been estimated to cause 1.5 million deaths and 21% of deaths worldwide in children under the age of 5 [4]. In India, most cases of diarrhea (98%) occurred during the first 2 years of life, peaking at 9–11 months [2]. Based on a literature review of studies published between 1986 and 1999 on childhood deaths caused by diarrhea and hospitalizations due to rotavirus, it was estimated that 440,000 annual deaths in children aged <5 years occur because of rotavirus infection [1]. An estimated 527,000 children aged <5 years died from rotavirus infection in 2004 with > 85% of these deaths in South-East Asia and sub-Saharan Africa [5]. Of 8929 Indonesian children in Purworejo district and Yogyakarta city hospitalized between August 2001 and April 2004, 16% presented with acute gastroenteritis, and 53% of the 1321 tested stool samples were positive for rotavirus [6].

This study involved children aged <5 years who were admitted with diarrhea in Sanglah Hospital, and the aims were to determine the prevalence of acute diarrhea and the rotavirus disease, and to determine the risk factors for rotavirus diarrhea.

## Methods

We conducted a prospective cohort study from April 2009 to December 2011 of hospitalized children with acute diarrhea. The study was a part of the study on Extension for Hospital-based Surveillance and Strain Characterization of Rotavirus Diarrhea Indonesia involving four hospitals throughout Indonesia as part of the Asian Rotavirus Surveillance Network.

For the sample size, we used a risk ratio of 1.1, α = 0.05, β = 0.2, and the proportion of individuals exposed to the outcome was 91%. The minimal sample size required was 494 patients. This study was approved by the Medical and Health Research Ethics Committee of the Faculty of Medicine, Gadjah Mada University, and parents’ written consent was obtained for participation in this study.

The study group included patients less than 5 years of age hospitalized with acute diarrhea. Patients admitted with diarrhea lasting more than 7 days and those with chronic diarrhea due to malabsorption and structural malformations were excluded from the study. We excluded diarrhea lasting more than 7 days because prolonged or persistent diarrhea are not usually caused by rotavirus.

The parents were questioned about the chief complaints and the degree of dehydration was examined. Parents were given a sterile screw-capped plastic container for collection of stool specimen within 24 hours after admission. A standardized clinical form was completed to obtain detailed information on the date of admission, age and sex of the patient, nutritional status, previous treatment, hydration status, symptoms, and final diagnosis.

For this study, we defined acute diarrhea as >3 loose stools within a 24-hour period [7] with a duration <7 days, and prolonged diarrhea was defined as an illness lasting between 7 and 14 days [8-10]. Vomiting was defined as the forceful expulsion of gastric contents at least once in a 24-hour period. Fever was defined by the presence of an axillary temperature greater than or equal to 38°C. If present, dehydration was graded as mild (dry mucus membranes, decreased tears and urine output), moderate (decreased skin turgor), or severe (excessively decreased skin turgor, tachycardia, low blood pressure, anuria, absence of tears) [11]. Breastfeeding was defined as provision of breast milk, either directly from the breast or expressed, and may constitute any portion of the infant’s diet.

A sufficient amount of whole stool specimen (5–10 ml) was collected for all admitted cases in a sterile screw-capped plastic container. Stool samples were divided in two sets: one was used for routine stool examination and the other was transported in refrigerated boxes to the Microbiology Laboratory at Gadjah Mada University, Faculty of Medicine, in Yogyakarta. It was stored at 4–8°C until laboratory testing for rotavirus. In the laboratory, the sample was aliquoted and stored at −70°C. Rotavirus was detected using an enzyme immunoassay for rotavirus (Dakopatts®, Dako Diagnostics Ltd., United Kingdom) in accordance with the manufacturer’s instructions.

Data were entered by a single investigator into the database that consists of information about clinical variables and rotavirus test results. Statistical analysis was performed using the SPSS 16.0 program. The final data were analyzed using the chi-square test and risk ratio to determine the significance of relevant parameters. The chi-square test was used with 95% confidence interval.

## Results

Patient demographics, clinical characteristics and laboratory findings are listed in Tables 1 and 2. Of the 656 cases, 577 (88.0%) had mild to moderate dehydration and 24 (3.7%) had severe dehydration. Two patients died and the case fatality rate was 0.3%. Of the 5805 patients below the age of 5 years who were admitted between April 2009 and December 2011, the prevalence of diarrhea was 11.3% and the prevalence of rotavirus infection in these hospitalized patients was 49.8%.

**Table 1:** Patient characteristics

**Characteristics**	**Total (n = 656)**
Sex, male, n (%)	400 (61.0)
Age, median (IQR) month	12.8 (7.5-20.4)
Weight, median (IQR) kg	8.5 (7-10)
Height, median (IQR) cm	75 (66-84)
Degree of dehydration, n (%)	
No dehydration	55 (8.4)
Mild-moderate	577 (88.0)
Severe	24 (3.7)
Length of stay, median (IQR) day	3 (3-4)
Rotavirus positive, n (%)	327 (49.8)

IQR = Interquartile range.

**Table 2:** Clinical manifestations in children with rotavirus and non-rotavirus diarrhea

**Variables**	**Rotavirus positive (n = 327)**	**Rotavirus negative (n = 329)**	**RR (95% CI), p value**
Chief complain, n (%)			
- Diarrhea	302 (92.4)	276 (83.9)	1.0 (Referent group)
- Vomiting	9 (2.8)	21 (6.4)	1.7 (1.00 to 3.02), 0.017
- Others	16 (4.9)	32 (9.7)	1.6 (1.04 to 2.36), 0.012
General condition at admission, n (%)			
- Well	155 (47.4)	150 (45.6)	1.0 (Referent group)
- Irritable	155 (47.4)	155 (47.1)	1.0 (0.87 to 1.19), 0.84
- Lethargy	17 (5.2)	24 (7.3)	1.2 (0.84 to 1.79), 0.26
Degree of dehydration, n (%)			
- No dehydration	17 (5.2)	38 (11.6)	1.0 (Referent group)
- Mild-moderate	299 (91.4)	278 (84.5)	0.6 (0.40 to 0.89), 0.03
- Severe	11 (3.4)	13 (4.0)	0.7 (0.38 to 1.21), 0.20
Vomiting, n (%)	275 (84.1)	247 (75.1)	1.4 (1.08 to 1.70), 0.004
Seizure, n (%)	23 (7.0)	30 (9.1)	0.9 (0.63 to 1.18), 0.33
Fever, n (%)	203 (62.1)	195 (59.3)	1.1 (0.90 to 1.24), 0.46
Bloody diarrhea, n (%)	1 (0.3)	7 (2.1)	4.0 (0.64 to 25.22), 0.07*
Outcome, n (%)			
- Recovered	319 (97.6)	319 (97.0)	1.0 (Referent group)
- Referral/Death	8 (2.4)	10 (3.0)	1.1 (0.67 to 1.90), 0.64

*Fisher’s exact test.

This study found an association between the occurrence of rotavirus and chief complain on admission, degree of dehydration, presence of vomiting (Table 2), and sex (Table 3), but no association was seen between rotavirus infection and nutritional status, breastfeeding, symptoms of seizure, fever, and hand washing behavior.

**Table 3:** Risk factors for children with rotavirus and non-rotavirus diarrhea

**Variables**	**Rotavirus positive (n = 327)**	**Rotavirus negative (n = 329)**	**RR (95% CI), p value**
Sex, Male, n (%)	214 (65.4)	186 (56.5)	1.2 (1.03 to 1.43), 0.019
Age, n (%) month			
0 – <3	19 (5.8)	22 (6.2)	1.0 (Referent group)
3 – <6	34 (10.4)	35 (10.6)	0.9 (0.63 to 1.41), 0.77
6 – <12	93 (28.4)	89 (27.1)	0.9 (0.63 to 1.30), 0.58
12 – <24	129 (39.4)	109 (33.1)	0.9 (0.60 to 1.21), 0.35
24 – <36	33 (10.1)	45 (13.7)	1.1 (0.72 to 1.67), 0.67
36 – <48	12 (3.7)	15 (4.6)	1.0 (0.61 to 1.78), 0.88
48 – <60	7 (2.1)	14 (4.3)	1.4 (0.70 to 2.77), 0.33
Nutritional status, n (%)			
Well-nourish	231 (70.6)	221 (67.2)	1.0 (Referent group)
Malnourish	96 (29.4)	108 (32.8)	1.1 (0.92 to 1.29), 0.34
Family member with diarrhea, n (%)	37 (11.3)	26 (7.9)	1.2 (0.96 to 1.50), 0.14
Eat by him/herself, n (%)	73 (22.3)	83 (25.2)	0.9 (0.76 to 1.11), 0.38
Fed by parent, n (%)	101 (30.9)	111 (33.7)	0.9 (0.79 to 1.11), 0.43
Breastfeeding, n (%)	125 (38.3)	122 (37.1)	1.0 (0.88 to 1.20), 0.76
Bottle-feeding, n (%)	245 (74.9)	245 (74.5)	1.0 (0.85 to 1.21), 0.89

A multivariate analysis showed that nutritional status, capability to eat by him/herself, fed by parent, breastfeeding, and bottle-feeding were protective factors for rotavirus diarrhea, but these results were not statistically significant (Table 4). This study showed that rotavirus diarrhea was more frequent in boys than girls (adjusted RR, 1.4; 95% CI, 1.04 to 1.97; p = 0.03).

**Table 4:** Multivariate analysis of risk factors for rotavirus diarrhea

**Variables**	**Regression coefficient**	**Adjusted RR (95% CI), p value**
Sex	0.361	1.4 (1.04 to 1.97), 0.03
Nutritional status	−0.117	0.9 (0.64 to 1.24), 0.50
Family member with diarrhea	0.404	1.5 (0.88 to 2.55), 0.14
Eat by him/herself	−0.100	0.9 (0.60 to 1.36), 0.63
Fed by parent	−0.094	0.9 (0.65 to 1.28), 0.59
Breastfeeding	−0.052	0.9 (0.68 to 1.33), 0.76
Bottle-feeding	−0.028	0.9 (0.66 to 1.44), 0.89

## Discussion

Diarrheal disease remains a significant health problem in many parts of the world [12]. Rotavirus is an important cause of diarrhea and contributes significantly to diarrhea in both developing and developed countries [13]. In Nepal, rotavirus accounts for up to 38% of diarrhea in children less than 5 years old [12]. In the 43 countries participating in the global surveillance network for rotavirus in 2009, 36% of hospitalizations for diarrhea among children aged <5 years were caused by rotavirus infection. Surveillance of the period between 2001 and 2008 revealed a detection rate of 40% in 35 countries with the virus distributed in similar regions [14]. The present study revealed that rotaviruses are important etiologic agents of acute diarrhea, accounting for 49.8% of all cases of acute gastroenteritis.

Rotavirus was detected in a higher proportion of males than females (RR, 1.2; 95% CI, 1.03 to 1.43; p = 0.019), indicating that males had a higher rotavirus stool excretion rate than females [3]. The male to female ratio of rotavirus infection was 1.6:1. This is in agreement with the finding that boys are twice more susceptible to rotavirus infection than girls and are more likely to be hospitalized [15]. Junaid et al. reported that the ratio was 1.8:1 in Nigeria, while Samir et al. and Puri et al. reported ratios of 1.5:1 and 1:2.4 for Bahrain and India, respectively [3]. According to the WHO scientific group [13,16], the number of affected males was up to 20% higher than the number of females in some studies, but it is not known whether this is due to a greater susceptibility to rotavirus exposure in boys or a greater likelihood of parents of affected boys seeking medical care.

According to the WHO scientific working group [13,16], most cases of rotavirus infections occur in children between 6 and 24 months with a peak incidence at 9 to 12 months. A surveillance conducted in 6 hospitals in 6 provinces of Indonesia found that children aged <3 months are less likely to have rotavirus diarrhea. Our findings confirmed that children aged <3 months are less susceptible to rotavirus, and children between 6 and 24 month are highly susceptible to rotavirus diarrhea.

Rotavirus is spread by direct person-to-person contact. Effective hand washing and disposal or disinfection of contaminated items are important measures in the prevention of rotavirus infection. Breastfeeding reduces gastrointestinal infections as breast milk contains lactadherin that is protective against symptomatic rotavirus infection. Lactadherin, secretory IgA, T-and B-lymphocytes, bactericidal lactoferrin, oligosaccharides and human milk glycans in breast milk protect the intestinal epithelium against pathogens. Human milk also contains anti-rotavirus antibodies that seem to play a smaller role against pathogens. Current WHO guidelines on the management of diarrhea recommend continued breastfeeding during a diarrhea episode [4].

One study asserted that transplacentally acquired immunoglobulin G antibodies and immunoglobulin A in breastmilk protect children aged <3 months against rotavirus infection. Human breast milk contains other components, including milk mucin, which have been shown to inhibit rotavirus replication and infection by binding to the virus [17,18].

A prospective study by Naficy et al. found a lower incidence of rotavirus diarrhea in infants that received breast milk [19]. Duffy et al. [20] followed a cohort of 197 infants and found no difference in rotavirus infection rates between the breast-fed and bottle-fed infants. Furthermore, a study by Wobudeya et al. [4] failed to show that breastfeeding was protective against rotavirus diarrhea in infants. Our study found no difference in rotavirus infection rates between bottle-fed and non-bottle-fed infants.

The close relationship between age, breastfeeding and rotavirus diarrhea could probably explain the observations in these reports. The peak age for rotavirus diarrhea is 6–11 months while the rates of breastfeeding begin to decline after 6 months. The protective effects of breastfeeding seem to wane with age [4]. This might explain why studies that focused on infants 6 months or younger tended to show the protective effect of breastfeeding.

**Figure 1:**
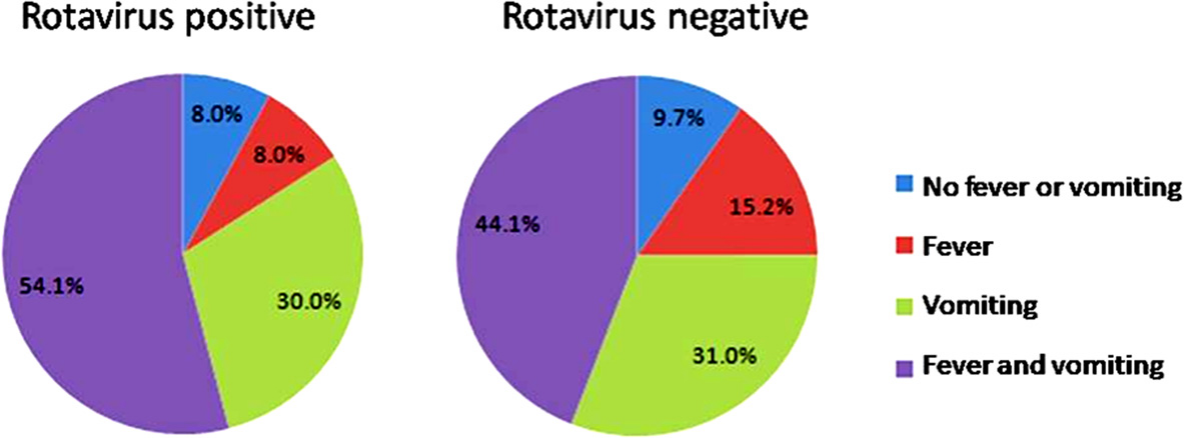
Comparison of symptoms of fever and vomiting between rotavirus and non-rotavirus diarrhea.

Vomiting appears to be more common in children with rotavirus diarrhea than those with non-rotavirus diarrhea [3]. The present study found that 83.3% of hospitalized children with rotavirus diarrhea presented with vomiting (RR, 1.4; 95% CI, 1.08 to 1.70, p = 0.004). The occurrence of the symptoms of diarrhea, fever and vomiting was significantly different between children with rotavirus diarrhea and those with non-rotavirus diarrhea [3]. The present study found that 54.1% of cases of rotavirus diarrhea presented with both fever and vomiting (Figure 1). These symptoms could increase the risk of dehydration and hospitalization. Although there appears to be an increasing trend in admitting young children with diarrhea, this appears to be associated with a low mortality, possibly because of better hospital clinical management [6].

**Figure 2:**
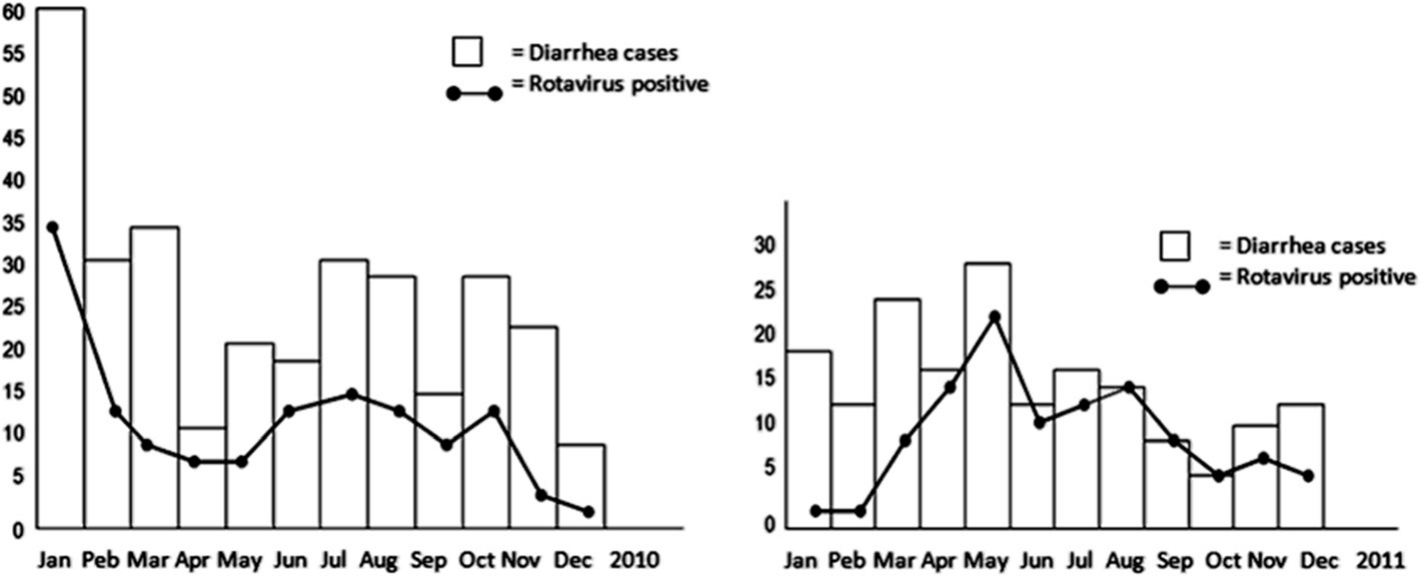
Seasonal variation in rotavirus positivity.

Rotavirus diarrhea occurs year round but there is an apparent increase in the prevalence of rotavirus infection in the cool, dry season. A study in Indonesia showed a clear seasonal trend of rotavirus infection in the hot and dry seasons with low humidity [6,17]. Another study in India found that 64.8% of rotavirus cases occurred in the cooler months i.e., from November to January. The present study found no consistent pattern in the number of rotavirus cases in 2 consecutive years (Figure 2). We assume that rotavirus diarrhea requiring hospital admission occur year round without any seasonal trend.

This study has several limitations, such as the stool specimen of the diarrhea cases were not cultured, so we did not know the characterization of the non-rotavirus diarrhea that may be consisting of both viral non-rotavirus and non-viral diarrhea. This study also did not include outpatient children; therefore we did not know the burden and risk factors for all rotavirus diarrhea in Denpasar.

## Conclusions

The prevalence of diarrhea at Sanglah Hospital was 11.3%. The prevalence of rotavirus diarrhea was 49.8%. The results of this study confirmed that diarrhea remains an important cause of hospitalization in children, and rotavirus was the most important etiology of diarrhea. The peak prevalence was between 6 and 24 months of age. We also found that boys were more susceptible to rotavirus infection than girls. Rotavirus diarrhea in hospitalized children was manifested as fever and vomiting more often than one symptom alone. In these children, good nutritional status and breastfeeding provided the same protection against rotavirus and non-rotavirus diarrhea.

## Abbreviations

IQR: Interquartile range; WHO: World Health Organization.

## Competing interests

The authors declare that they have no competing interests.

## Authors’ contributions

All authors read and approved the final manuscript. HS participated in the design of the study, data collection, statistical analysis, and helped draft the manuscript. SB and YS participated in the design of the study, data collection, and helped draft the manuscript. IPGK participated in data collection and helped draft the manuscript. IGNSP participated in the design of the study and helped draft the manuscript.

## Authors’ information

HS is the Master of Biomedicine and pediatric researcher in the Department of Child Health, Medical School, Udayana University/Sanglah Hospital.

IPGK and IGNSP are pediatric gastroenterology consultants and researchers in the Department of Child Health, Medical School, Udayana University/Sanglah Hospital.

SB is the Professor of Child Growth and Development – Social Pediatrics in the Department of Child Health, Medical School, Udayana University/Sanglah Hospital.

YS is the Professor of Pediatric Gastroenterology in the Department of Child Health, Medical School, Gadjah Mada University/Sardjito Hospital. She is the project head of Extension for Hospital-based Surveillance and Strain Characterization of Rotavirus Diarrhea Indonesia.

## Pre-publication history

The pre-publication history for this paper can be accessed here:

http://www.biomedcentral.com/1471-230X/14/54/prepub
